# Serum Syndecan-1: a potent prognostic biomarker for transplant outcomes

**DOI:** 10.3389/fmed.2026.1828404

**Published:** 2026-05-14

**Authors:** Takayuki Yokokawa, Masahiko Fukatsu, Koichiro Fukuchi, Yuki Sato, Takahiro Sano, Daisuke Koyama, Satoshi Kimura, Miki Furukawa, Shingo Yamada, Takayuki Ikezoe

**Affiliations:** 1 Department of Hematology, Fukushima Medical University, Fukushima, Japan; 2 Department of Hematology, North Fukushima Medical Center, Date, Japan; 3 R&D Center, Shino-Test Corporation, Sagamihara, Japan

**Keywords:** biomarker, early mortality, endothelial, hematopoietic stem cell transplantation, Syndecan-1

## Abstract

**Introduction:**

Post-transplant–associated complications arising from endothelial injury, such as sinusoidal obstruction syndrome (SOS) and transplant-associated thrombotic microangiopathy (TA-TMA), are associated with high mortality rates following hematopoietic stem cell transplantation (HSCT). Syndecan-1 (SDC1), a cell-surface proteoglycan released into the circulation upon endothelial damage, is a potential biomarker for vascular integrity. We previously demonstrated that elevated serum SDC1 levels were associated with disease severity and poor prognosis in patients with COVID-19.

**Methods:**

This prospective study investigated whether longitudinal serum SDC1 kinetics could predict early mortality in HSCT recipients; serial blood samples were collected from 130 patients undergoing hematopoietic stem cell transplantation (HSCT), from the day of HSCT through day 28 post-transplant, to investigate whether serum SDC1 levels could serve as a predictive marker for early mortality within 100 days after HSCT.

**Results:**

During the observation period, 12 patients died, of whom 9 deaths were attributed to conditions associated with endothelial injury, including SOS (*n* = 2), TA-TMA (*n* = 2), and idiopathic pneumonia syndrome (*n* = 3). Notably, serum SDC1 levels at Days 0, 14, and 21 after HSCT were significantly higher in patients who died within 100 days than in those who survived beyond Day 100.

**Conclusion:**

These findings suggest that serum SDC1 has the potential to predict early post-transplant mortality and warrants further validation in larger prospective studies.

## 1 Introduction

Allogeneic hematopoietic stem cell transplantation (HSCT) remains the only curative treatment option for various hematologic malignancies and refractory hematopoietic disorders, regardless of whether they are congenital or acquired. HSCT is a complex therapeutic modality that involves the combined use of various agents, including cytotoxic chemotherapy, antimicrobial drugs, immunosuppressants, and radiation. Recent advances in medical technology have broadened the indications for HSCT, and its safety has improved due to refinements in conditioning regimens and acute graft-versus-host disease (aGVHD) prophylaxis (1, 2). However, the development of severe complications arising from endothelial cell injury remains a major factor that compromises post-transplant survival.

These severe post-HSCT complications include sinusoidal obstruction syndrome (SOS) and transplant-associated thrombotic microangiopathy (TA-TMA), which are now recognized as a spectrum of diseases with a common underlying pathomechanism: systemic endothelial cell injury, termed “endothelial syndrome” (3). One of the initial steps in this syndrome is the destruction of the endothelial glycocalyx, the innermost lining of the vasculature that plays a critical role in maintaining vascular barrier function and suppressing thrombosis and inflammation (4, 5). HSCT-associated endothelial syndrome includes not only SOS and TA-TMA, but also capillary leak syndrome, idiopathic pneumonia syndrome (IPS), and aGVHD (3).

The Syndecan (SDC) family consists of four transmembrane heparan sulfate proteoglycans (SDC1-4) that play crucial roles in cell signaling and adhesion (6). Each SDC is composed of three distinct domains: an extracellular domain, a transmembrane domain, and a cytoplasmic domain. Through glycosaminoglycan chains such as heparan sulfate and chondroitin sulfate attached to the extracellular domain, SDCs interact with a multitude of ligands, including growth factors, cytokines, and extracellular matrix (ECM) proteins (6, 7). Each member of the SDC family exhibits a distinct tissue distribution. SDC1 is predominantly expressed in epithelial cells and plasma cells, whereas SDC2 is found in mesenchymal tissues, liver, and neuronal cells. SDC3 is primarily located in neuronal cells, while SDC4 is known to be ubiquitously expressed in various cells throughout the body (7–9).

Among these, SDC1 is a key component of the glycocalyx covering vascular endothelial cells and plays an essential role in maintaining vascular homeostasis, in part by regulating cell adhesion through its interaction with integrins (10). As a key component of the endothelial glycocalyx, SDC1 is known to be involved in the suppression of intravascular inflammation and the regulation of leukocyte trafficking (11). Under stressful conditions such as inflammation or tissue injury, enzymes known as sheddases, including matrix metalloproteinases (MMPs), cleave SDC1 from the cell surface (10). This process, termed “shedding,” releases the SDC1 ectodomain into the circulation as soluble SDC1. Soluble SDC1 is not merely cellular debris but is known to act as a functional molecule that can, for instance, amplify inflammatory responses (12). Therefore, elevated serum levels of SDC1 are considered a sensitive biomarker reflecting the extent of systemic cellular damage, including that of vascular endothelial cells. We have developed an enzyme-linked immunosorbent assay (ELISA) to measure human SDC1 and demonstrated that elevated serum SDC1 levels are associated with decreased platelet counts and increased mortality in septic patients (13). In addition, we recently found that increased serum levels of SDC1 are associated with disease severity and predictive of mortality in COVID-19 patients (8).

The present study investigated the potential of serum SDC1 levels to predict the development of treatment-related complications and mortality by Day 100 after transplantation, and to assess whether this could ultimately help reduce mortality from these complications.

## 2 Materials and methods

### 2.1 Study population and sample collection

This study included 217 patients who underwent allogeneic HSCT at the Department of Hematology, Fukushima Medical University Hospital between 2017 and 2024. Written informed consent and serial blood samples were obtained from 148 participants. Blood samples were collected at five time points: on the day of transplantation (Day 0), and weekly on days 7, 14, 21, and 28 after transplantation.

Serum levels of SDC1 were measured using enzyme-linked immunosorbent assays (ELISA) developed by Shino-Test Corporation (Kanagawa, Japan), as previously described (14).

This study was approved by the Institutional Review Board of Fukushima Medical University.

### 2.2 Diagnosis of complications

Diagnosis and evaluation of the severity of SOS were based on the criteria published by the European Society for Blood and Marrow Transplantation (EBMT) (15). For the diagnosis of TA-TMA, we utilized the international harmonization criteria (16); however, the measurement of soluble C5b-9 is not routinely available in Japan. Therefore, the diagnosis of TA-TMA was based on the harmonization criteria excluding C5b-9, which have been widely adopted in the recent study (17). Patients were diagnosed with TA-TMA if at least four criteria of the harmonization criteria were met, excluding the measurement of C5b-9. To ensure diagnostic consistency, all TA-TMA cases were reviewed independently by two hematologists (SF and TI) with expertise in post-transplant complications, and discordant cases were adjudicated through consensus discussion. IPS was diagnosed when imaging studies revealed diffuse pulmonary infiltrates in the absence of an infectious etiology and heart failure. The diagnosis of CLS was based on a previous publication (18). The diagnosis and the grade of aGVHD were determined according to the criteria issued by Mount Sinai Acute GVHD International Consortium (19).

### 2.3 Conditioning regimens

Conditioning regimens were classified into myeloablative conditioning (MAC) and reduced-intensity conditioning (RIC) according to the criteria proposed by Bacigalupo et al. (20).

Specifically, the regimens were classified as follows:

- MAC (myeloablative conditioning): Regimens expected to cause irreversible pancytopenia without stem cell rescue. Examples in our cohort include TBI ≥ 8 Gy or busulfan > 8 mg/kg.
- RIC (reduced-intensity conditioning): Regimens that cause varying degrees of cytopenia but are not myeloablative, typically incorporating a dose reduction of at least 30% in TBI or alkylating agents compared to MAC.

### 2.4 Statistical analysis

The impact of biomarker levels on patient prognosis was also assessed. An AUC > 0.7 was considered to indicate “clinically acceptable discriminatory power,” based on the general interpretation that an AUC of 0.5 indicates no discrimination, 0.7–0.8 is acceptable, 0.8–0.9 is excellent, and >0.9 is outstanding (21). This threshold has been widely adopted in previous clinical studies utilizing similar statistical analyses to define the adequacy of diagnostic or prognostic model (22). In this study, Biomarkers achieving an AUC > 0.7 were regarded as clinically significant.

To identify independent prognostic factors for Day 100 mortality, univariate and multivariate Cox proportional hazards analyses were performed. Variables that showed statistical significance in the univariate analysis were included in the initial multivariate model. A backward stepwise elimination method was then applied to select the final set of independent predictors. Hazard ratios (HR) and 95% confidence intervals (CI) were calculated for each factor.

These were analyzed using EZR (Easy R, Version 1.62; Saitama Medical Center, Jichi Medical University, Saitama, Japan) (23).

## 3 Results

### 3.1 Study population and patient characteristics

**Figure 1:**
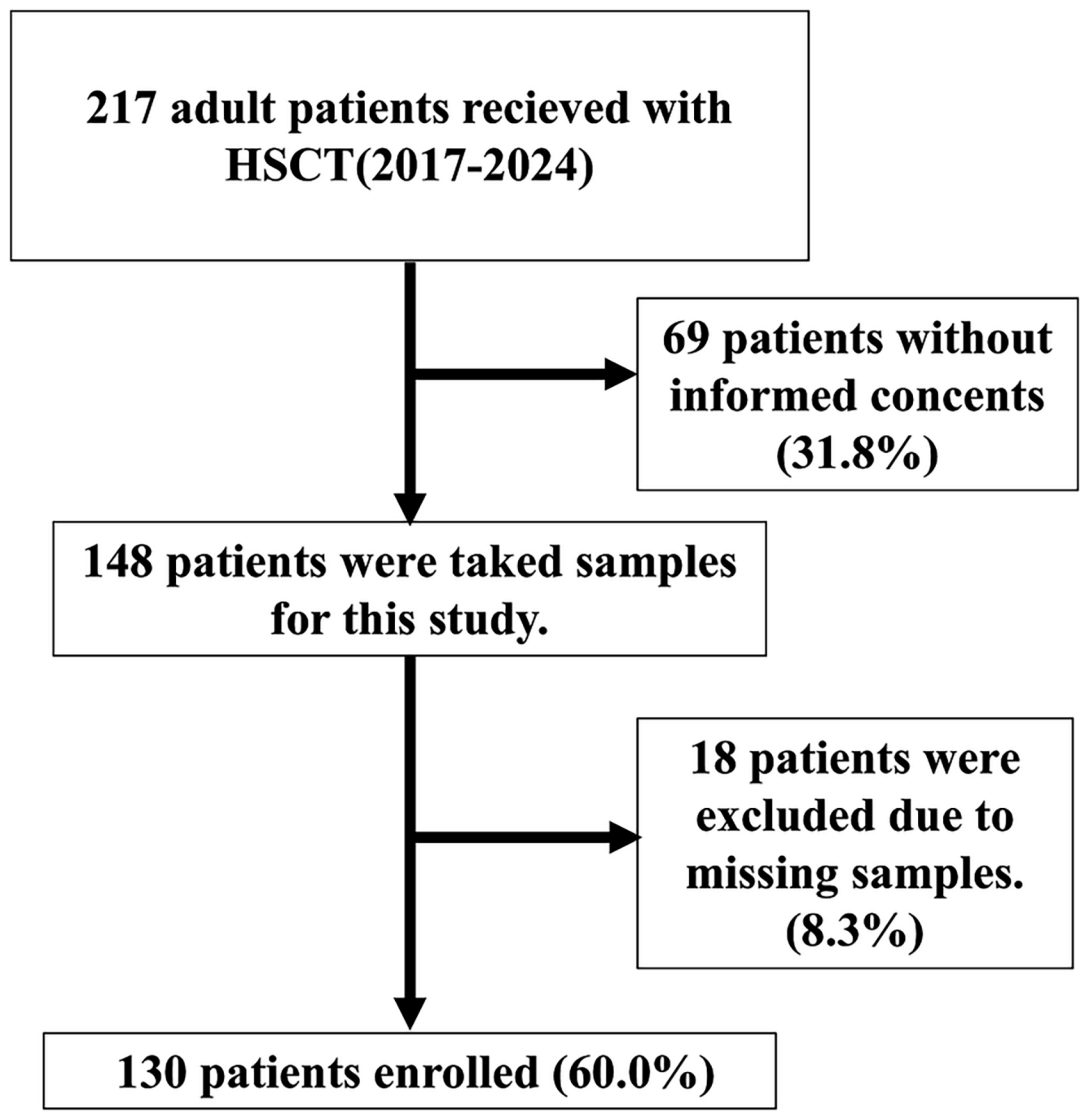
Patient flowchart. Of the 217 adult patients who underwent allogeneic HSCT at our institution between 2017 and 2024, 69 were excluded because informed consent was not obtained. Samples were collected from the remaining 148 patients. Of these, a further 18 patients were excluded from the final analysis due to missing or incomplete samples, resulting in a final study cohort of 130 patients.

Between 2017 and 2024, a total of 217 adult patients underwent HSCT at our institution. Written informed consent was obtained from 148 patients. Of these, a further 18 patients were excluded from the final analysis due to missing or incomplete blood samples, resulting in a final study cohort of 130 patients. The flowchart is shown in Figure 1. Therefore, the present study comprises these 130 consented patients. Detailed patient characteristics are summarized in Table 1.

**Table 1:** Patient’s characteristics.

Characteristics	All patients (*n* = 130)
Age (years), median (range)	55 (18–68)
Sex	
Male, *n* (%) Female, *n* (%)	66 (50.8) 64 (49.2)
Underlying disease	
AML, *n* (%) ALL, *n* (%) MDS, *n* (%) AA, *n* (%) CML, *n* (%) Other, *n* (%)	51 (39.2) 27 (20.8) 24 (18.5) 6 (4.6) 5 (3.8) 17 (13.1)
Complications	
None, *n* (%) SOS, *n* (%) TA-TMA, *n* (%) IPS aGVHD Grade III/IV, *n* (%)	100 (76.9) 10 (7.7) 14 (10.8) 5 (3.8) 15 (11.5)
aGVHD	
Developed aGVHD, *n* (%) Grade I/II Grade III/IV	69 (53.1) 46 (35.4) 15 (11.5)
Conditioning regimen	
MAC, *n* (%) RIC, *n* (%)	68 (52.3) 62 (47.7)
GVHD prophylaxis	
FK base, *n* (%) Cya base, *n* (%)	130 (100) 0 (0)
Donor source	
CBT, *n* (%) PBSCT, *n* (%) BMT, *n* (%)	41 (31.5) 62 (47.6) 27 (20.8)
Prior HSCT	
N, *n* (%) Y, *n* (%)	117(90.0) 13(10.0)

AML, acute myeloid leukemia; ALL, acute lymphoblastic leukemia; MDS, myelodysplastic syndromes; AA, aplastic anemia; CML, chronic myeloid leukemia; ML, malignant lymphoma; SOS, sinusoidal obstruction syndrome; TA-TMA, transplant-associated thrombotic microangiopathy; aGVHD, acute graft-versus-host disease; HSCT, hematopoietic stem cell transplantation.

The median age of the cohort was 55 years (range, 18–68 years), and it included 66 males and 64 females. The most common underlying disease was acute myeloid leukemia, accounting for 51 cases.

Regarding complications, 14 patients developed TA-TMA, with a median onset at day 61 (range, 8–169), and 10 patients developed SOS, with a median onset at day 35 (range, 8–61). aGVHD occurred in 69 patients, of whom 15 (11.5%) developed Grade III/IV aGVHD. Three patients developed IPS, which resulted in a fatal outcome. The median observation period in this cohort was 329.5 days (range, 4– 2,553 days).

Among the 30 patients who developed endothelial complications, there was an overlap of 3 patients who were diagnosed with both SOS and TA-TMA.

Regarding the overlap with aGVHD Grade III/IV, 4 of the 10 patients with SOS (40%) also developed acute GVHD Grade III/IV, while 7 of the 14 patients with TA-TMA (50%) also developed aGVHD Grade III/IV. On the other hand, five patients developed IPS, and no other endothelial complications occurred.

In this cohort, among the 10 patients who developed SOS, 8 patients were treated with defibrotide according to institutional guidelines.

### 3.2 Causes of death within 100 days post-HSCT

Twelve patients died within 100 days post-HSCT. The causes of death and the clinical characteristics of these patients are summarized in Table 2.

**Table 2:** Characteristics of patients who died within 100 days post-HSCT.

	Age Sex	Disease	Donor source	SDC1 of Day 0 (reference value 4.1)	aGVHD prophylaxis	Prior HSCT	aGVHD, Grade & Day	SOS	TA-TMA	Day of death	Cause of death
								Severity	Risk		
1	54 F	NHL	rPBSCT	1.9	FK	Y	III Day9	N	Y	Day18	TA-TMA aGVHD
								n.a	High		
2	29 M	B-ALL	CBT	15.9	FK	Y	N	N	N	Day7	Sepsis MOF
								n.a	n.a		
3	54\u00B0F	HL	rPBSCT	33.6	FK+MMF	N	N	N	N	Day4	Multiorgan hemorrhage IPS
								n.a	n.a		
4	55 M	MDS EB-2	CBT	3.5	FK+MMF	N	III Day52	Y	N	Day85	aGVHD Severe infection
								Very Severe	n.a		
5	59 M	MDS EB-2	CBT	18.6	FK+MMF	N	N	N	N	Day33	IPS
								n.a	n.a		
6	51\u00B0F	AML	rPBSCT	9.3	PTCY+FK+MMF	N	N	Y	N	Day88	SOS Cerebral infarction
								Very Severe	n.a		
7	65 M	B-ALL	rPBSCT	3.7	PTCY+FK+MMF	N	N	N	N	Day44	IPS
								n.a	n.a		
8	62 M	AML	rPBSCT	5.6	PTCY+FK+MMF	N	N	N	N	Day11	AHF
								n.a	n.a		
9	65 M	MDS EB-1	CBT	6.6	FK+MMF	N	III Day51	N	Y	Day63	TA-TMA MOF ARDS
								n.a	High		
10	62 M	AML	rPBSCT	7.7	PTCY+FK+MMF	N	N	N	N	Day22	Disease Progression DIC MOF
								n.a	n.a		
11	62 M	MDS RAEB1	uBMT	4.2	FK+sMTX	N	II Day21	N	N	Day43	Disease progression
								n.a	n.a		
12	56F	B-ALL	CBT	4.5	FK+MMF	N	N	N	N	Day6	Cardiogenic shock Septic shock
								n.a	n.a		

SDC1, Syndecan-1; AML, acute myeloid leukemia; MDS, myelodysplastic syndromes; EB, excess blasts; RAEB1, refractory anemia with excess of blasts-1; ALCL, anaplastic large cell lymphoma; B-ALL, B-cell acute lymphocytic leukemia; HL, Hodgkin lymphoma; NHL, non-Hodgkin lymphoma; CBT, cord blood transplantation; rPBSCT, related peripheral blood stem cell transplantation; uPBSCT, unrelated peripheral blood stem cell transplantation; uBMT, unrelated bone marrow transplantation; FK, tacrolimus; MMF, mycophenolate mofetil; sMTX, short-term methotrexate; PTCY, post-transplant cyclophosphamide; aGVHD, acute graft-versus-host disease; TA-TMA, transplant-associated thrombotic microangiopathy; SOS, sinusoidal obstruction syndrome; HPS, hemophagocytic syndrome; IPS, idiopathic pneumonia syndrome; AHF, acute heart failure; ARDS, acute respiratory distress syndrome; DIC, disseminated intravascular coagulation; MOF, multi-organ failure; N, no; Y, yes; n.a, not assessed.

Of the 12 patients, seven died directly from endothelial syndrome-related complications, including SOS (*n* = 1), TA-TMA (*n* = 2), Grade III/IV aGVHD (*n* = 1), and IPS (*n* = 3). Two patients died of sepsis on days six and seven, respectively. The remaining two patients died from the progression of AML and MDS, respectively. Another patient died of acute heart failure on day 11 after HLA-haploidentical HSCT incorporating post-transplant cyclophosphamide.

### 3.3 Patients who died by Day 100 after HSCT had significantly higher serum levels of SDC1

**Figure 2:**
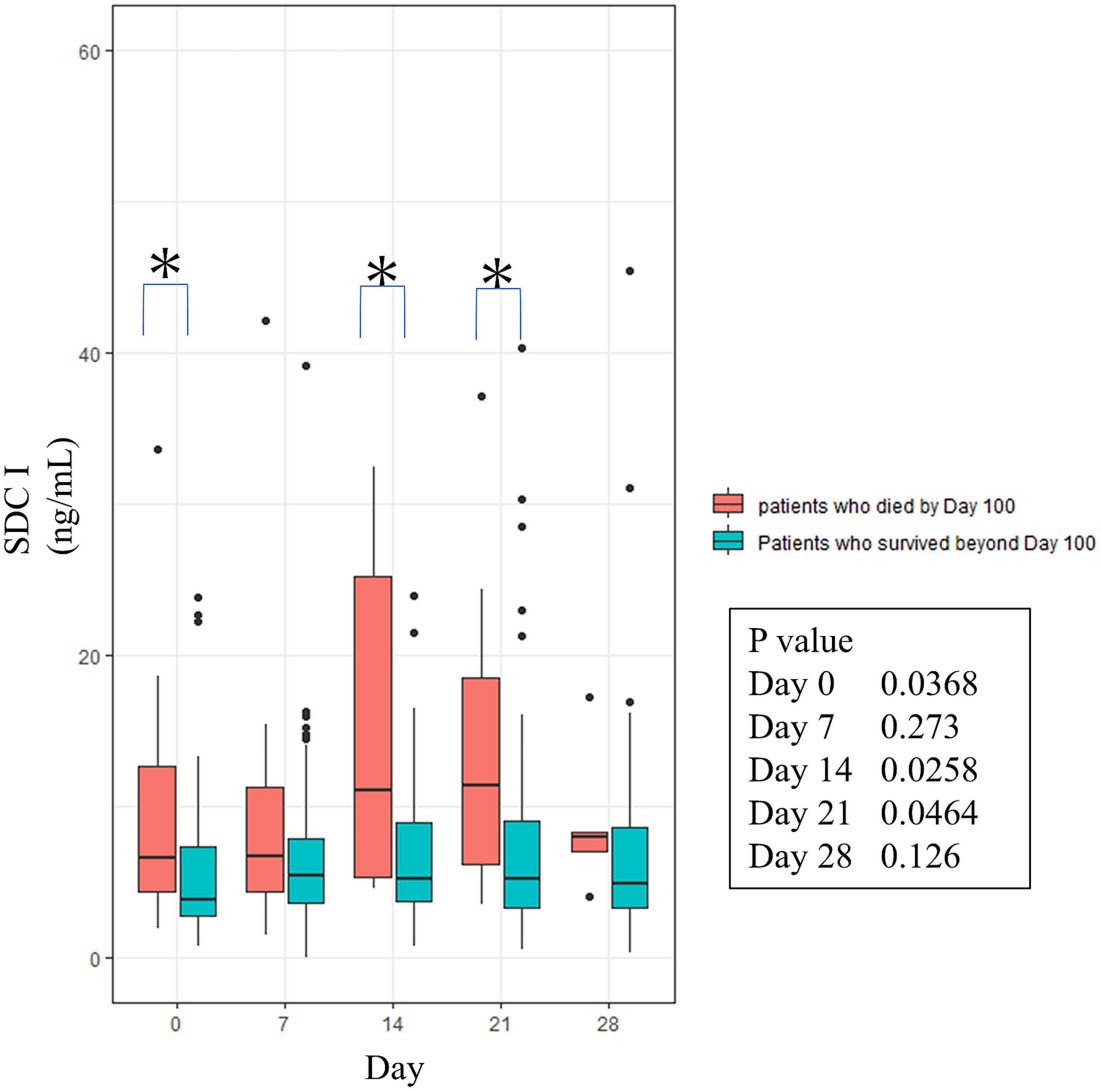
Association between longitudinal changes in serum SDC1 and the development of severe post-transplant complications. Controls were defined as patients who remained free from SOS and TA-TMA. The figures show the changes in serum SDC1 concentrations over time, stratified into two groups: patients who survived beyond Day 100, and patients who died by Day 100. *, Indicates a statistically significant difference. SDC1, Syndecan-1; SOS, sinusoidal obstruction syndrome; TA-TMA, transplant-associated thrombotic microangiopathy.

Next, we measured the longitudinal kinetics of serum SDC1 levels, comparing patients who died within 100 days post-HSCT (early mortality group) with those who survived beyond this period. Significant differences between the two groups were observed at Days 0, 14, and 21, which are shown in Figure 2.

Our previous study found that the median level of SDC-1 in healthy volunteers (*n* = 8; median age, 32 years) was 2.6 ng/mL (range, 1.8–3.8 ng/mL) (8), which was significantly higher than that in HSCT recipients at Day 0 (median, 4.1 ng/mL).

**Figure 3:**
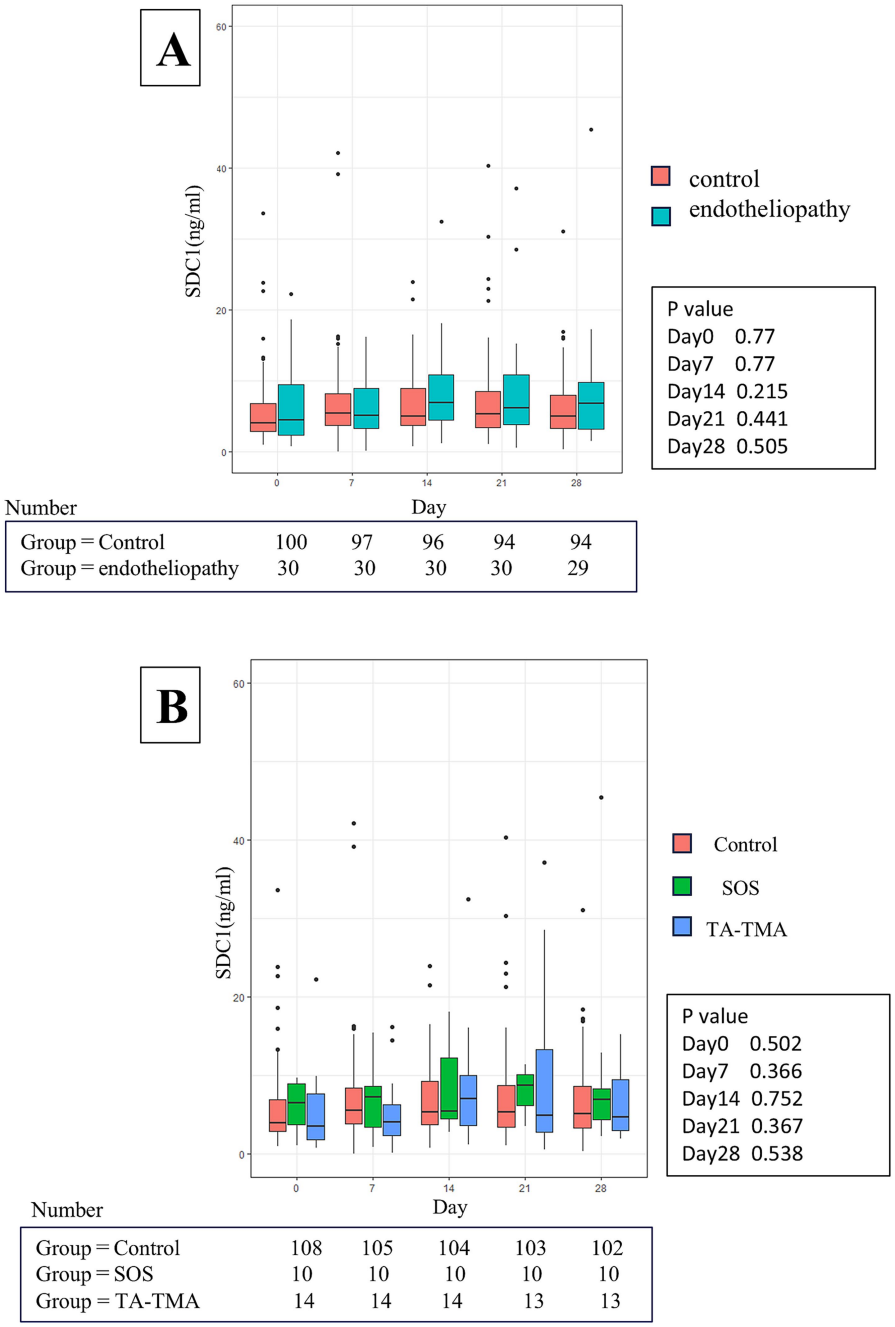
Longitudinal changes in serum SDC1 level. The control group consisted of patients who did not develop any endothelial complications, including SOS, TA-TMA, IPS, and grade III–IV acute GVHD. **(A)** The figures show the changes in serum SDC1 levels over time, stratified into two groups: control and endotheliopathy. **(B)** The figures show the changes in serum SDC1 levels over time, stratified into three groups: control, SOS, and TA-TMA. SDC1, Syndecan-1; SOS, sinusoidal obstruction syndrome; TA-TMA, transplant-associated thrombotic microangiopathy; IPS, idiopathic pneumonia syndrome; aGVHD, acute graft-versus-host disease.

Subsequently, the 130 patients were divided into two groups: the endothelia syndrome group (patients who developed SOS, TA-TMA, IPS, or grade III/IV aGVHD) and the control group (patients who developed none of these complications) (Figure 3A). Contrary to our expectations, there was no significant difference in SDC1 levels between the two groups at any time point. Further analyses also found no statistically significant difference in SDC1 levels among individual complications, including SOS and TA-TMA (Figure 2B). However, interestingly, the SOS group tended to have higher serum SDC1 levels than the control group at most time points (Figure 3B).

### 3.4 SDC1 predicts early mortality

Based on these findings, we then performed ROC curve analyses for each of these significant time points to determine their predictive ability and optimal cutoff values. Consistent with the findings in Figure 1, the ROC curve analyses for Days 14, 21 yielded significant results (AUC > 0.7, *p* < 0.05). In addition, Day 28 showed significant results, but Day 0’s AUC was under 0.7.

**Figure 4:**
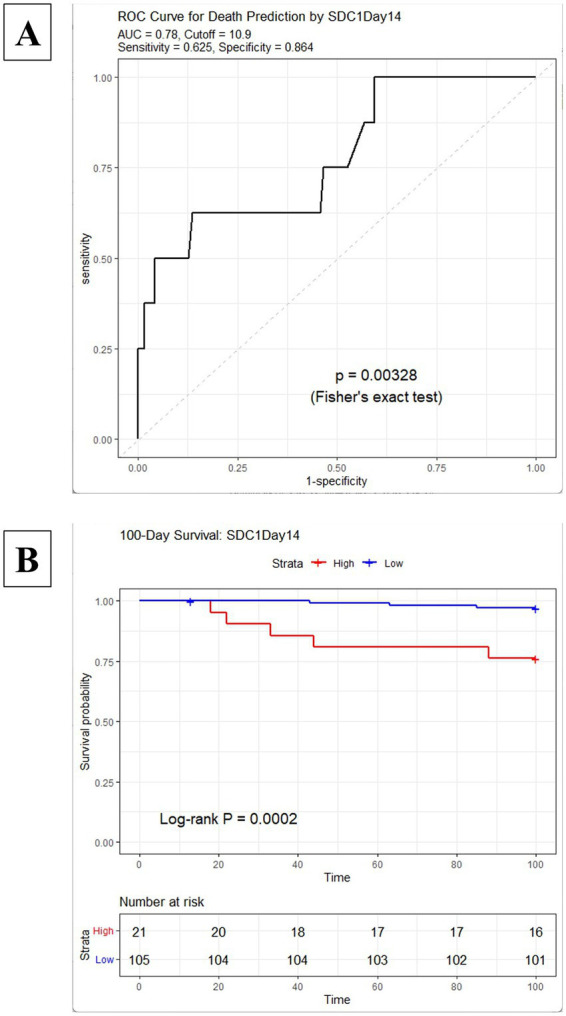
ROC curve and Kaplan–Meier survival analysis based on the Day 14 cutoff. Kaplan–Meier curves for the survival in patients stratified by optimal cutoff values for Day 7. *p*-values were calculated using the log-rank test. The analysis was performed for: **(A)** ROC curve at Day 7; **(B)** Kaplan–Meier curves stratified by the Day 7 cutoff value. SDC1, Syndecan-1; ROC, receiver operating characteristic.

To further assess the clinical relevance of these findings, Kaplan–Meier analysis was performed for the survival curve. Patients were stratified into high- and low-marker groups based on the optimal SDC1 cutoff values for the all-time points. At each of these time points, patients with high SDC1 levels (above the cutoff) had a significantly higher incidence of mortality within 100 days compared to those with low levels. Interestingly, a significant difference in survival was also observed when using the cutoff value from Days 0 and 7, even though its AUC did not exceed 0.7 in the ROC analysis (Supplementary Figures 1, 2). The results for Day 14, which demonstrated the highest predictive ability, are presented in Figure 4.

### 3.5 Univariate analysis and multivariate analysis

In the univariate analysis, elevated SDC1 levels at each time point (except Day 0) and the development of grade III–IV aGVHD were significantly associated with Day 100 mortality (Tables 3, 4). In the multivariate analysis, both high SDC1 levels and grade III–IV aGVHD remained significant independent risk factors at most time points (Table 5). Notably, at Day 28, a high SDC1 level was the sole independent predictor of mortality. Conversely, at Day 0, only grade III–IV aGVHD remained significant, and SDC1 did not show independent prognostic value at this specific time point.

**Table 3:** Univariate analysis of pretransplant risk factors for the Day 100 mortality.

Variables	*n*	Death within Day 100 Incidence (%)	HR (95% CI)	*p*
Age, y				
<50 ≧50	47 83	1 (2.13) 11 (13.3)	References 7.028 (1.301–130.6)	0.066
Sex				
Male Female	66 64	8 (12.1) 4 (6.25)	References 0.483 (0.124–1.622)	0.256
Prior HSCT				
N Y	117 13	10 (8.55) 2 (15.4)	References 1.945 (0.276–8.669)	0.426
Donor source				
CBT PBSCT BMT	41 62 27	5 (12.2) 6 (9.7) 1 (3.7)	References 2.786 (0.444–54.0) 3.611 (0.540–71.4)	0.354 0.254
Conditioning				
RIC MAC	62 68	8 (12.9) 4 (5.88)	References 0.422 (0.108–1.416)	0.177
Diseases				
AML ALL MDS AA CML Other	51 27 24 6 5 17	3 (5.88) 3 (11.1) 3 (12.5) 0 (0) 0 (0) 2 (11. 8)	References 2 3.2 - - 2.13	0.417 0.150 0.995 0.996 0.430
aGVHD GradeIII/IV				
N Y	105 15	8 (7.6) 4 (26.7)	References 4.86 (1.15–18.33)	0.022
Year of HSCT				
2017–2021 2022–2024	53 77	4 (7.55) 8 (10.4)	References 1.42 (0.42–5.56)	0.584

BMT, bone marrow transplantation; PBSCT, peripheral blood stem cell transplantation; CBT, cord blood transplantation; RIC, reduced-intensity conditioning; MAC, myeloablative conditioning; AML, acute myeloid leukemia; ALL, acute lymphoblastic leukemia; MDS, myelodysplastic syndromes; AA, aplastic anemia; CML, chronic myeloid leukemia; aGVHD, acute graft-versus-host disease; HSCT, hematopoietic stem cell transplantation; N, no; Y, yes.

**Table 4:** Univariate analysis of cutoff values derived from ROC curves for the Day 100 mortality.

Point of cut-off	HR (95%CI)	*p*
SDC1 Day0		
Low High	References 6.78 (1.26–126)	0.071
SDC1 Day7		
Low High	References 5.21 (1.33–20.5)	0.015
SDC1 Day14		
Low High	References 10.6 (2.38–56.0)	0.002
SDC1 Day21		
Low High	References 8.68 (1.97–45.3)	0.005
SDC1 Day28		
Low High	References 10.5 (1.63–205)	0.034

SDC1, Syndecan-1.

**Table 5:** Multivariate analysis with backward variable selection.

Day	Factors	HR (95%CI)	*p*
Day 0	aGVHD Grade III/IV		
	N Y	References 4.86 (1.26–18.8)	0.022
Day 7	aGVHD Grade III/IV		
	N Y	References 14.9 (2.48–90.1)	0.0032
	SDC1 Day7		
	Low High	References 11.2 (2.04–61.8)	0.0055
Day 14	aGVHD Grade III/IV		
	N Y	References 14.1 (2.25–88.1)	0.0047
	SDC1 Day14		
	Low High	References 14.6 (2.46–86.6)	0.0032
Day 21	aGVHD Grade III/IV		
	N Y	References 14.9 (2.4–92.8)	0.0038
	SDC1 Day21		
	Low High	References 12.7 (2.15–75.6)	0.0051
Day 28	SDC1 Day28		
	Low High	References 10.5 (1.19–93.3)	0.0344

aGVHD, acute graft-versus-host disease; SDC1, Syndecan-1.

## 4 Discussion

Of the 130 patients in this study, a total of 12 patients died within 100 days after transplantation. Nine out of 12 patients developed transplant-associated endothelial syndrome (TA-TMA, *n* = 2; SOS, *n* = 1; grade III/IV aGVHD, *n* = 1; IPS, *n* = 3), which resulted in fatal outcomes. Other causes of death included sepsis (*n* = 2), which is also associated with endothelial dysfunction via systemic inflammation and cytokine storms (13). Collectively, nine out of 12 cases of early mortality were associated with endothelial dysfunction. Importantly, serum SDC1 levels in seven of nine patients on Day 0 exceeded the control group’s reference values (Table 2).

Numerous studies have investigated the role of SDC1 in various pathological conditions. In the pediatric setting, authors reported on a cohort of 113 patients who underwent HSCT. They demonstrated that serum SDC1 levels were significantly elevated in patients who developed SOS or steroid-refractory aGVHD (24). On a mechanistic level, previous studies in a murine model reported that the loss of SDC1 from the endothelium promotes a pro-inflammatory vascular phenotype (7). However, to the best of our knowledge, no previous studies have serially monitored serum SDC1 concentrations and investigated the association between their longitudinal changes and early mortality due to post-transplant endothelial injury. This underscores the novelty and significance of the present study.

In this study cohort, a total of three patients died within the very early post-transplant period (by Day 7). As shown in Table 2, all three patients (cases 2, 3 and 12) exhibited elevated serum SDC1 levels on Day 0 compared to the median Day 0 SDC1 levels of the entire cohort. This observation raises the possibility that an association between elevated serum SDC1 and subsequent mortality may exist as early as Day 0. To explore this hypothesis, we performed an exploratory analysis of the predictive value of Day 0 SDC1 for early mortality. The ROC curve and the corresponding Kaplan–Meier curve are presented in Supplementary Figure 1. While the ROC analysis did not reach the threshold for clinical significance (AUC = 0.66, *p* = 0.058), the Kaplan–Meier analysis, using the cutoff value derived from this ROC curve, did show a significant separation in survival between the high- and low-SDC1 groups (Supplementary Figure 1). This discrepancy suggests that even if the AUC from the ROC curve does not exceed the conventional threshold of 0.7, serum SDC1 levels on the day of transplantation might still hold clinical utility for predicting very early mortality. Indeed, the longitudinal data presented in Figure 2 also show a significant result towards a difference in SDC1 concentrations on Day 0 between patients who died within 100 days and those who survived. It is therefore plausible that a similar analysis in a larger cohort could yield more clinically and statistically significant results.

We will now consider the clinical implications of our findings. This study demonstrated that an increase in serum SDC1 levels is associated with early post-transplant mortality due to endothelial injury. We showed that monitoring SDC1 levels from Day 14 onwards could enable the prediction of this poor outcome. This finding holds the potential to broaden therapeutic options, such as modulating the dosing of calcineurin inhibitors, which are toxic to vascular endothelium, or introducing anti-inflammatory interventions at an earlier stage.

Furthermore, although our analysis of Day 0 SDC1 levels did not reach statistical significance, it did suggest a potential predictive capability. A re-evaluation of therapeutic strategies at such an early time point could be crucial for reducing mortality from transplant-related complications and ultimately improving transplant outcomes. A key area for future investigation is the predictive value of pre-transplant SDC1 levels, which we were unable to assess in the present study. If the prognostic value of pre-transplant SDC1 levels for early mortality is validated in future prospective studies, it could offer a novel tool for risk stratification. For instance, identifying patients with high SDC1 as a “high-risk” population before transplantation might allow for the implementation of enhanced supportive care strategies or the enrollment of these patients into clinical trials investigating endothelium-protective agents. This approach could represent a new paradigm in personalized transplant medicine, moving towards risk-adapted supportive care.

In the present study, 12 patients died within the early post-transplant period (up to Day 100). However, three of these deaths were attributed to causes unrelated to endothelial injury. To further investigate the specific association between SDC1 dynamics and endothelial-related complications, we performed a sub-analysis excluding these three cases. In this refined cohort of 127 patients, both the ROC curve analysis and Kaplan–Meier survival analysis were recalculated to ensure the robustness and specificity of SDC1 as a prognostic biomarker for endothelial-driven mortality. As a result, statistically significant differences were observed specifically at Day 14 (AUC = 0.80, cutoff = 10.9) and Day 28 (AUC = 0.752, cutoff = 6.9). Also, these days demonstrated significant prognostic value in the Kaplan–Meier survival analysis (Supplementary Figures 3, 4). Regarding the other time points, the AUC values for Day 0 (AUC = 0.677, Cut off = 14.6) and Day 21 (AUC = 0.692, Cut off = 12.4) were below 0.7 in the ROC curve analysis; however, the Kaplan–Meier analysis demonstrated that SDC1 levels at these time points could still significantly predict early mortality (Supplementary Figure 5). In contrast, no significant results were obtained for Day 7 in either analysis.

We also explored the association between SDC1 levels and other endothelial-related syndromes, specifically engraftment syndrome (ES) and capillary leak syndrome (CLS), whose prognoses are relatively favorable. ES was diagnosed based on Spitzer’s criteria (25), which include fever, rash, and pulmonary edema without evidence of infection. CLS was identified by the presence of the classic clinical triad (hypotension, hemoconcentration, and hypoalbuminemia) as previously described (26).

Although the number of patients who fulfilled the diagnostic criteria was small (ES, *n* = 3; CLS, *n* = 0), we observed a higher tendency of serum SDC1 levels in patients with ES compared to those without (Supplementary Figure 6). This finding is consistent with the pathophysiology of ES, which involves cytokine-mediated acute endothelial injury during the recovery of neutrophils.

We chose Day 100 mortality for several important, interconnected reasons. First, Day 100 mortality is a well-established endpoint for evaluating early, non-relapse mortality driven by regimen-related toxicities and acute complications like the endothelial syndromes under investigation. Our primary hypothesis was that Syndecan-1, as a marker of acute endothelial injury, would be most strongly associated with these early, toxicity-related events. Second, and more pragmatically, our study’s sampling protocol was designed to capture biomarker dynamics during this critical early phase, with serial measurements taken only up to Day 28 post-HSCT. This sampling timeframe is well-suited to predict early outcomes like Day 100 mortality, but it inherently limits our ability to robustly predict late-occurring events. A single, early biomarker measurement is less likely to have a sustained predictive power for late mortality, which is often driven by different factors such as disease relapse or chronic GVHD that evolve over a longer period. Therefore, focusing on Day 100 mortality was not only the most appropriate approach to test our specific hypothesis, but also the most scientifically rigorous choice given the temporal limitations of our biomarker data.

This study has several limitations. First is the lack of pre-transplantation data. Given that some patients died very early in the post-transplant period, a longitudinal analysis including pre-transplant time points would be necessary to enhance the reliability of the results presented in this study. Second, this was a single-center study with a relatively small number of samples. It may have limited the statistical power to detect subtle differences across all time points. Second, the cohort included diverse transplant attributes, such as different conditioning regimens and GVHD prophylaxis, which could potentially influence SDC1 dynamics. Therefore, further research involving a larger dataset from a multicenter collaboration is warranted.

## 5 Conclusion

In conclusion, our study identifies post-transplant serum SDC1 as a promising prognostic biomarker for early, all-cause mortality after allogeneic HSCT. While its direct association with specific endothelial syndromes was not established in our cohort, its strong link to mortality underscores the critical importance of systemic endothelial health in the early post-transplant period. Although our findings do not support the use of SDC1 to guide specific therapies at this stage, they highlight its potential role in risk stratification for identifying patients who may require more intensive monitoring. Further large-scale, prospective studies are warranted to validate these findings and to elucidate the precise biological mechanisms underlying this association.

## Data Availability

The raw data supporting the conclusions of this article will be made available by the authors, without undue reservation.
