# Supplementary figures and images for: Serum Syndecan-1: a potent prognostic biomarker for transplant outcomes
Source: Front Med (Lausanne). 2026 May 14;13:1828404. doi: 10.3389/fmed.2026.1828404 (PMC13216469; doi:10.3389/fmed.2026.1828404)

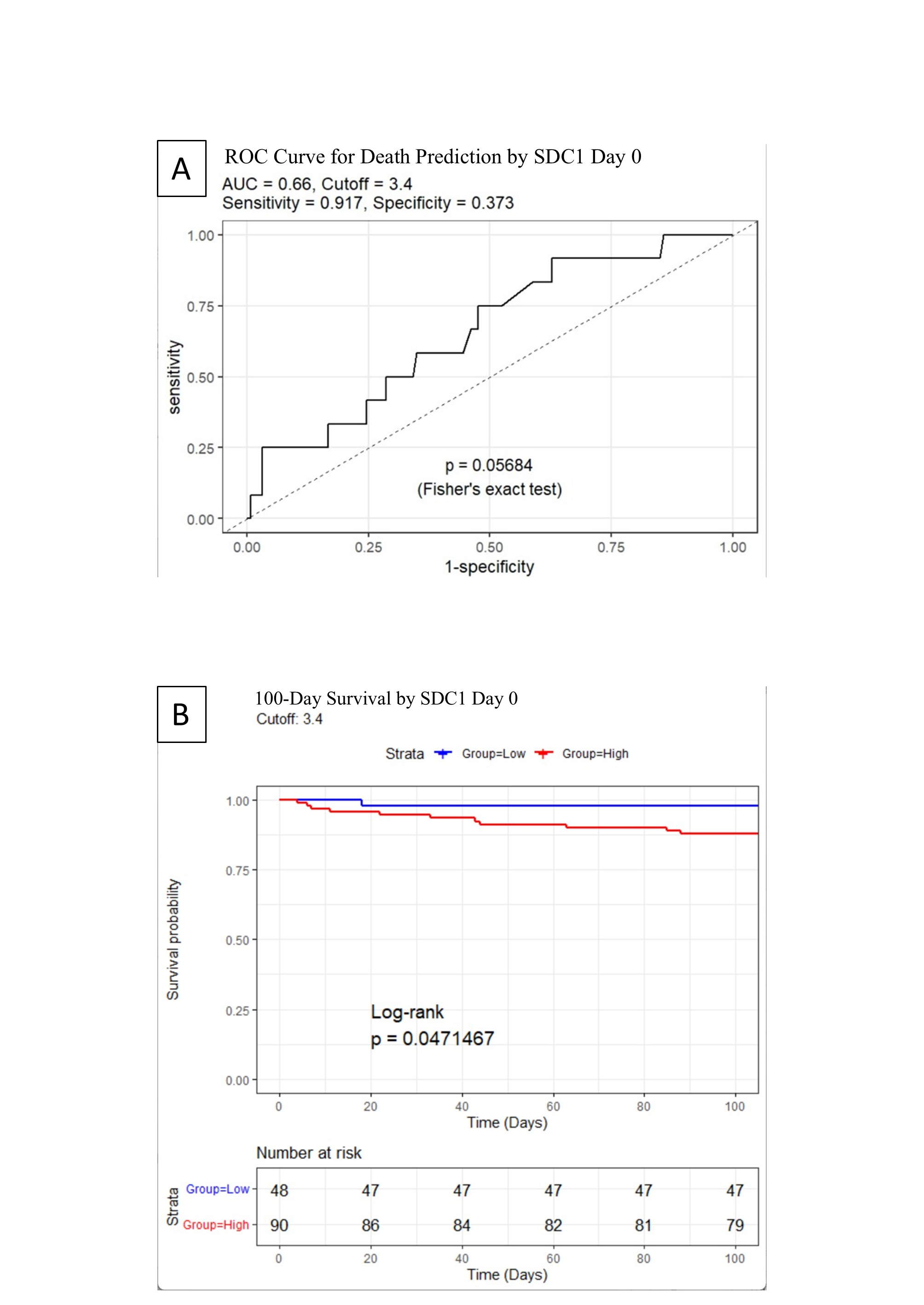

Supplement: Supplementary file 1 [file Image_1.TIF]

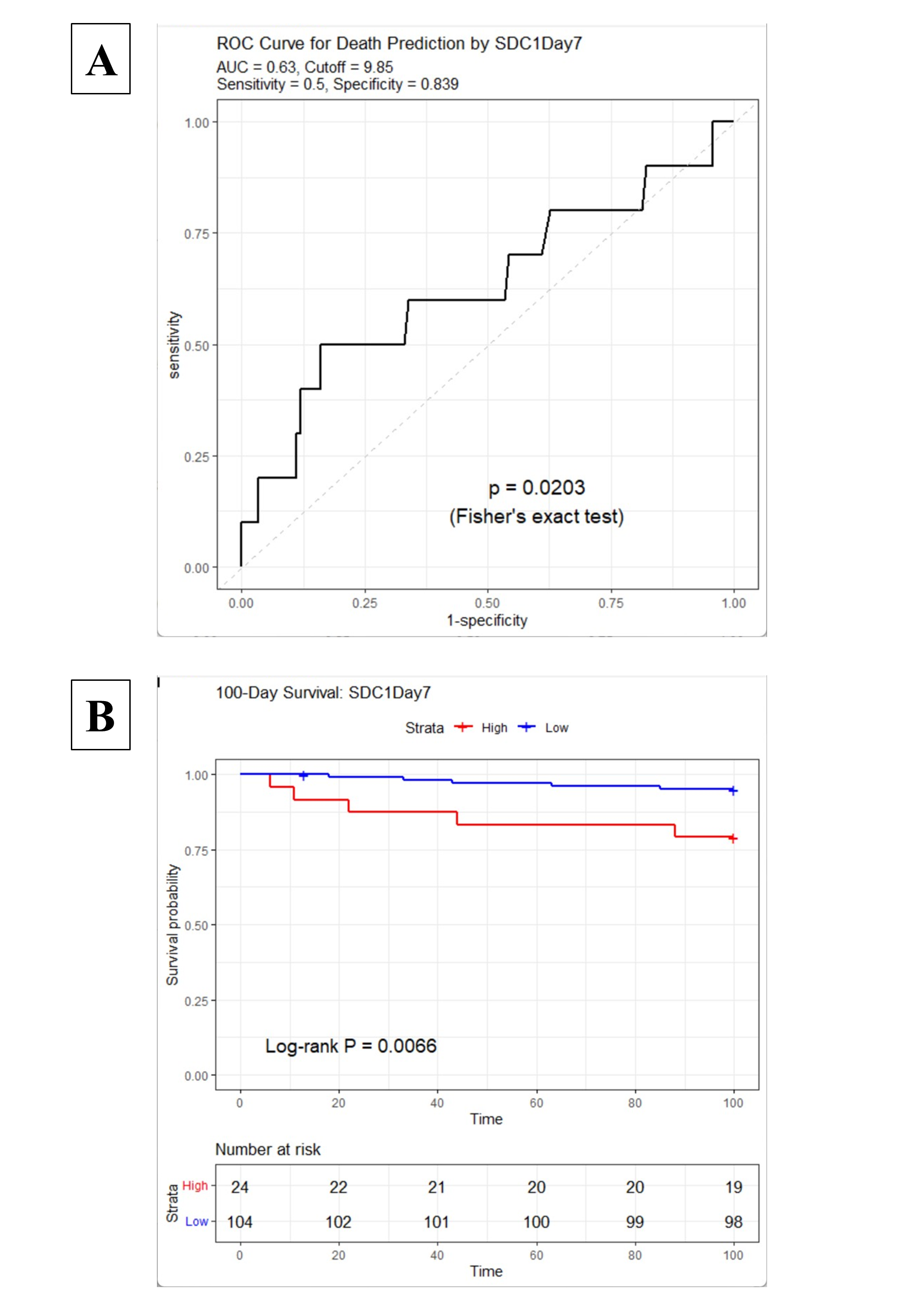

Supplement: Supplementary file 2 [file Image_2.TIF]

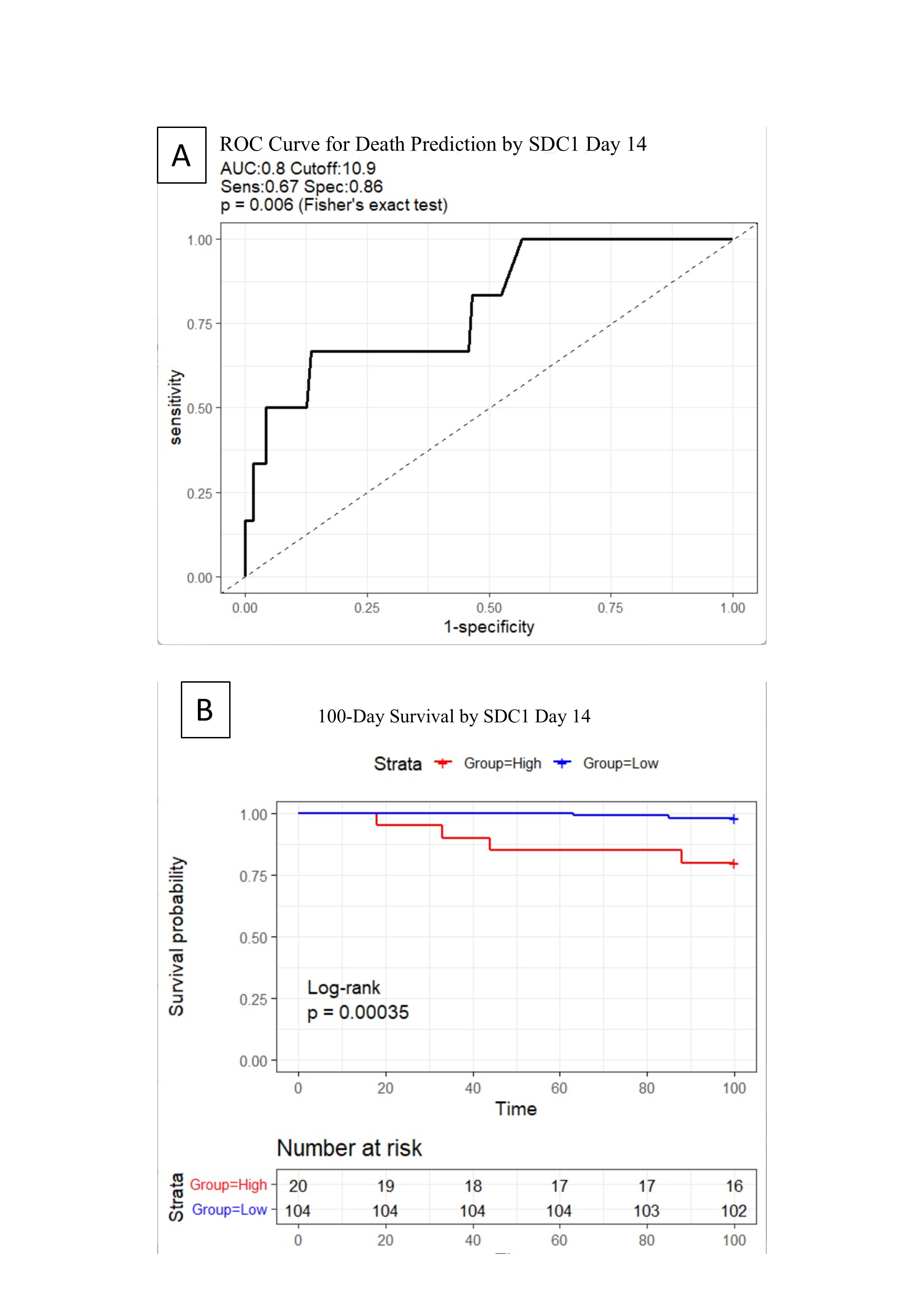

Supplement: Supplementary file 3 [file Image_3.TIF]

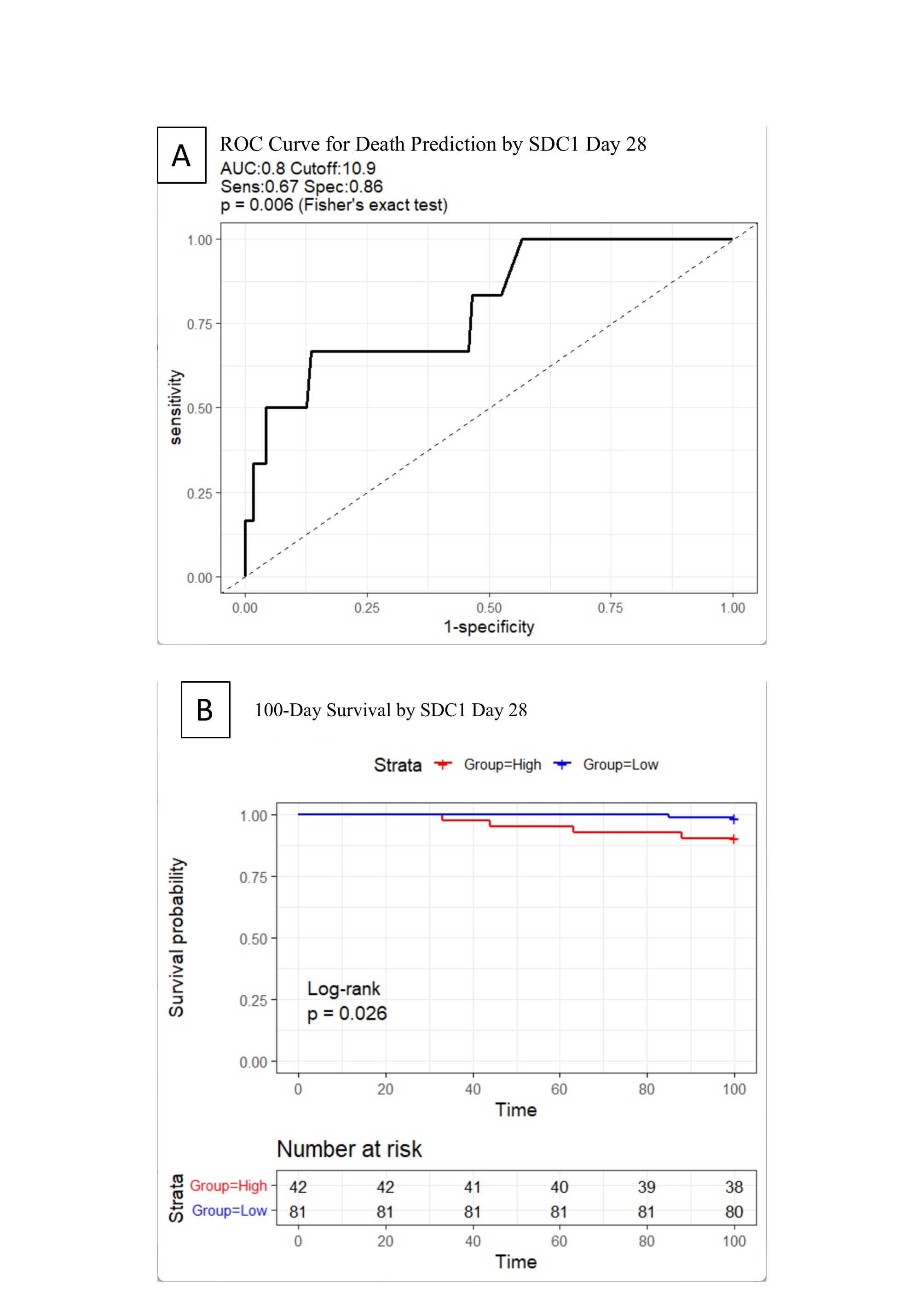

Supplement: Supplementary file 4 [file Image_4.TIF]

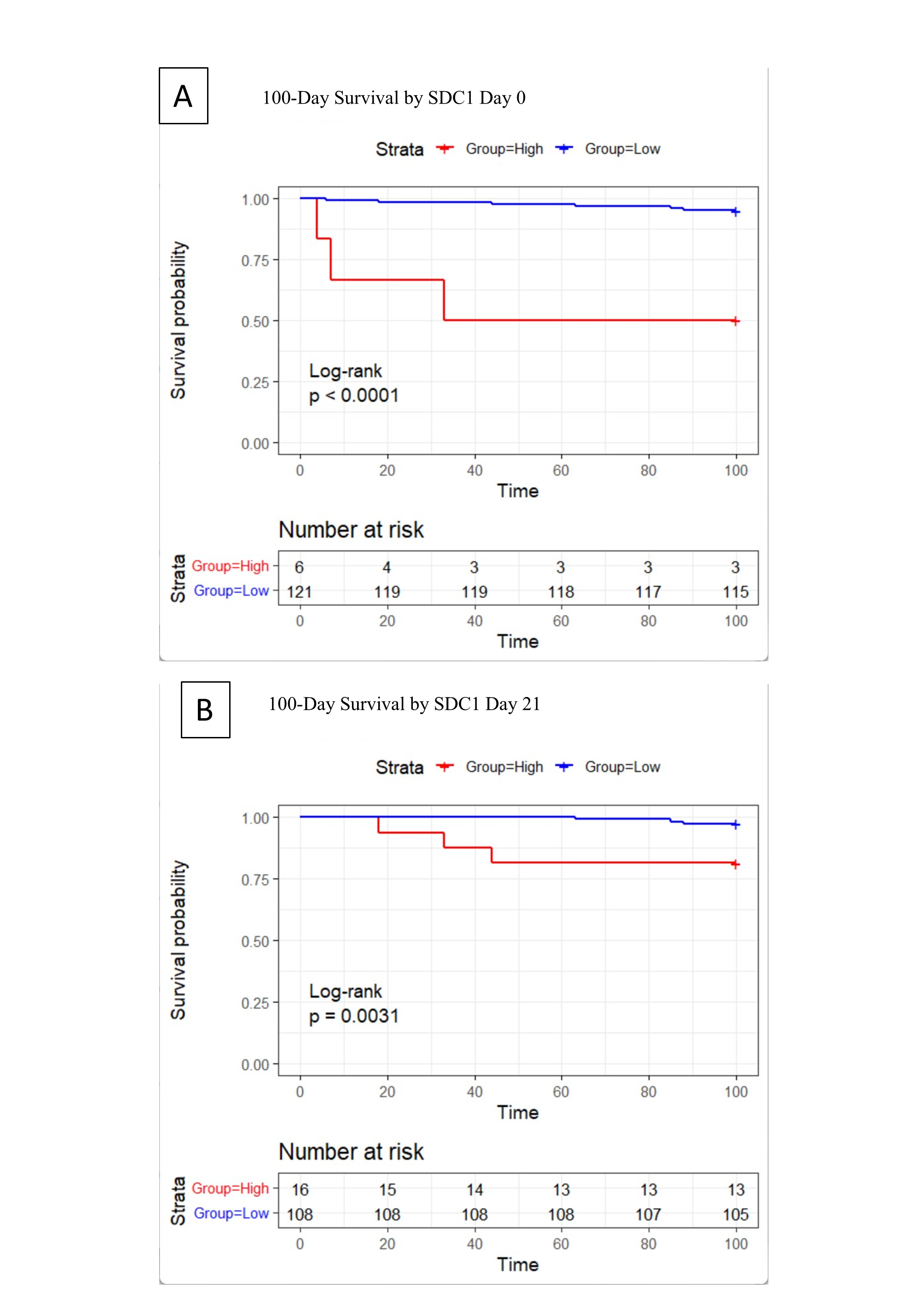

Supplement: Supplementary file 5 [file Image_5.TIF]

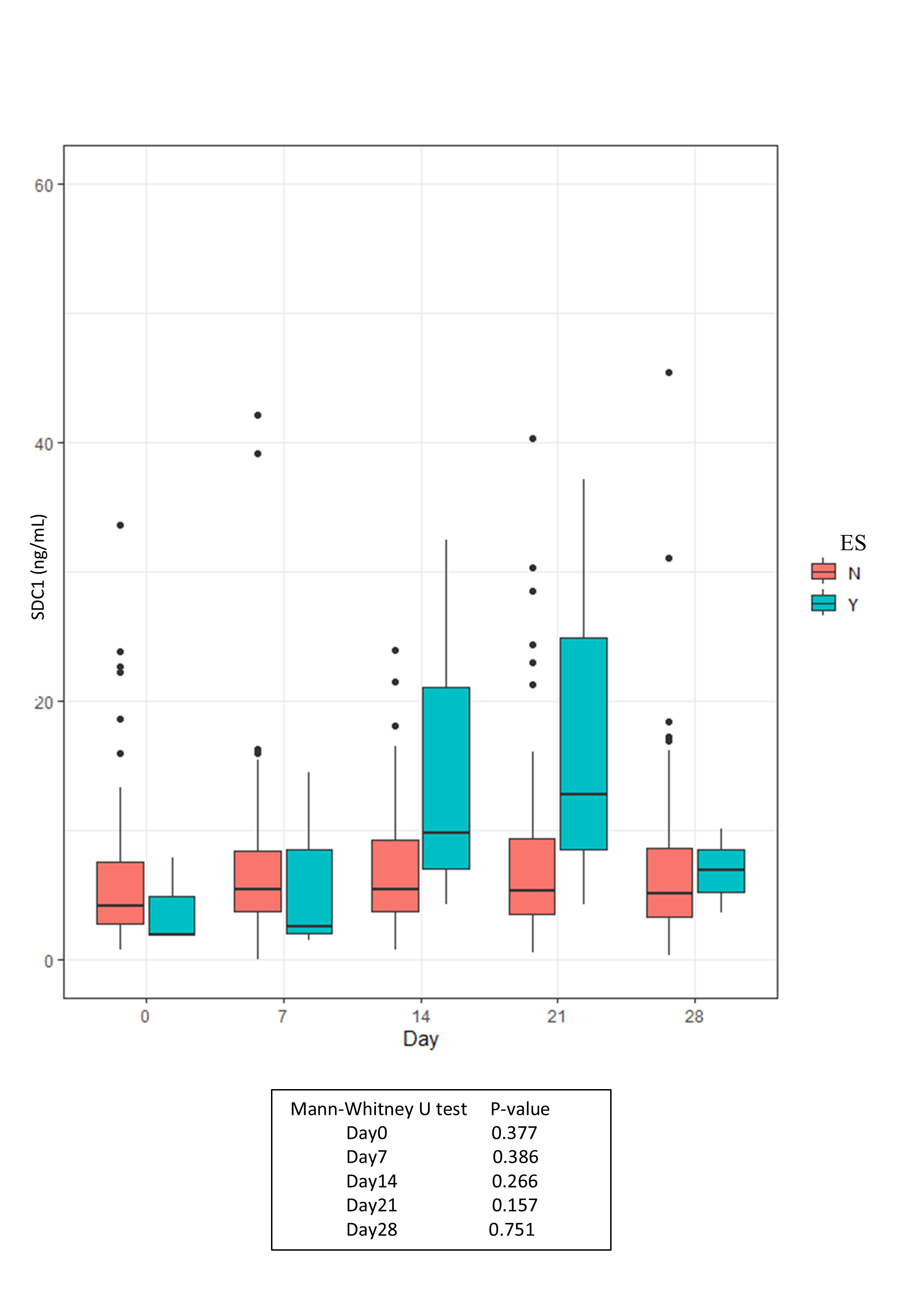

Supplement: Supplementary file 6 [file Image_6.TIF]
