# Supplementary material for: Serum Syndecan-1: a potent prognostic biomarker for transplant outcomes
Source: Front Med (Lausanne). 2026 May 14;13:1828404. doi: 10.3389/fmed.2026.1828404 (PMC13216469; doi:10.3389/fmed.2026.1828404)
Supplement: Supplementary file 7 [file Table_1.DOCX]

**Serum Syndecan-1: A Potent Prognostic Biomarker for Transplant Outcomes**

**Takayuki Yokokawa¹, Masahiko Fukatsu¹, Koichiro Fukuchi¹, Yuki Sato¹, Takahiro Sano¹, Daisuke Koyama¹, Satoshi Kimura¹, Miki Furukawa^1,2^, Shingo Yamada^3^, Takayuki Ikezoe¹**
¹ Department of Hematology, Fukushima Medical University
² Department of Hematology, North Fukushima Medical Center

^3^ R&D Center, Shino-Test Corporation, Sagamihara, Japan.

Address correspondence: Takayuki Ikezoe, MD., PhD.

Department of Hematology, Fukushima Medical University.

Hikarigaoka-1, Fukushima City, Fukushima 960-1295, Japan.

**e-mail:** [**ikezoet@fmu.ac.jp**](mailto:ikezoet@fmu.ac.jp)

**Key words:** hematopoietic stem cell transplantation, Syndecan-1, endothelial, early mortality, biomarker

**Figure Legend**

**Supplementary Figure 1.** **ROC Curve and Kaplan-Meier Survival Analysis Based on the Day 0 Cutoff**

Kaplan-Meier curves for the survival in patients stratified by optimal cutoff values for Day 0. P-values were calculated using the log-rank test. The analysis was performed for:

(a) ROC curve at Day 0

(b) Kaplan-Meier curves stratified by the Day 0 cutoff value

SDC1: syndecan-1, ROC: receiver operating characteristic

**Supplementary Figure 2.** **ROC Curve and Kaplan-Meier Survival Analysis Based on the Day 7 Cutoff**

Kaplan-Meier curves for the survival in patients stratified by optimal cutoff values for Day 0. P-values were calculated using the log-rank test. The analysis was performed for:

(a) ROC curve at Day 7

(b) Kaplan-Meier curves stratified by the Day 7 cutoff value

SDC1: syndecan-1, ROC: receiver operating characteristic

**Supplementary Figure 3.** **sub-analysis of ROC and Kaplan-Meier Survival Analysis Based on the Day 14 Cutoff**

Kaplan-Meier curves for the survival in patients stratified by optimal cutoff values for Day 14. P-values were calculated using the log-rank test. The analysis was performed for:

(a) ROC curve at Day 14

(b) Kaplan-Meier curves stratified by the Day 14 cutoff value

SDC1: syndecan-1, ROC: receiver operating characteristic

**Supplementary Figure 4.** **sub-analysis of ROC and Kaplan-Meier Survival Analysis Based on the Day 28 Cutoff**

Kaplan-Meier curves for the survival in patients stratified by optimal cutoff values for Day 28. P-values were calculated using the log-rank test. The analysis was performed for:

(a) ROC curve at Day 28

(b) Kaplan-Meier curves stratified by the Day 28 cutoff value

SDC1: syndecan-1, ROC: receiver operating characteristic

**Supplementary Figure 5.** **sub-analysis of Kaplan-Meier Survival Analysis**

Kaplan-Meier curves for the survival in patients stratified by optimal cutoff values for Day 28. P-values were calculated using the log-rank test. The analysis was performed for:

(a) Kaplan-Meier curves stratified by the Day 0 cutoff value

(b) Kaplan-Meier curves stratified by the Day 21 cutoff value

**Supplementary Figure 6: Comparison of SDC1 levels between patients with or without ES**

Patients were categorized into two groups based on whether they suffered from ESor not, and the longitudinal changes in SDC1 levels were compared between these groups.

SDC1: syndecan-1, ES: engraftment syndrome
